# A Pilot Randomized Control Study on Effect Brief Heart Rate Variability Biofeedback as a Complementary Treatment in Men with Methamphetamine Use Disorder

**DOI:** 10.3390/ijerph19095230

**Published:** 2022-04-25

**Authors:** Cheng-Fang Yen, Chih-Hung Ko, Chih-Yao Hsu, Hung-Chi Wu, Yu-Yi Yang, Peng-Wei Wang

**Affiliations:** 1 Department of Psychiatry, School of Medicine, College of Medicine, Kaohsiung Medical University, Kaohsiung 80708, Taiwan; chfaye@kmu.edu.tw (C.-F.Y.); cyberkoseed@gmail.com (C.-H.K.); iugyyy@gmail.com (Y.-Y.Y.); 2 Department of Psychiatry, Kaohsiung Medical University Hospital, Kaohsiung 80708, Taiwan; 3 Department of Psychiatry, Kaohsiung Municipal Hsiao-Kang Hospital, Kaohsiung Medical University, Kaohsiung 80708, Taiwan; 4 Department of Addiction Science, Kai-Suan Psychiatric Hospital, Kaohsiung 80276, Taiwan; oldtype@gmail.com (C.-Y.H.); wuhcmail@gmail.com (H.-C.W.)

**Keywords:** HRV, methamphetamine, addiction, craving, substance use disorder

## Abstract

The aims of this study were to investigate the efficacy of heart rate variability biofeedback (HRVBFB) intervention in terms of reducing craving, severity of dependence, and rate of positive methamphetamine urine testing in men taking part in a methamphetamine use disorder outpatient treatment program. Sixty-one adult men received either HRVBFB treatment plus treatment as usual (TAU) over four weeks or TAU only. Men receiving HRVBFB showed significantly greater reductions in craving, dependence severity, and the rate of positive methamphetamine urine testing at the end of the intervention and four weeks of follow-up. The analyses further showed that the levels of craving and dependence severity at treatment entry were predictive of changes in craving and dependence severity at the end of treatment and follow-up, respectively. The baseline status of a positive methamphetamine urine test only predicted a positive methamphetamine urine test at the end of treatment, not at the end of the follow-up period. Our results showed HRVBFB intervention has merits as an adjunct treatment to ameliorate cravings and reduce the severity of dependence experienced by persons with methamphetamine use disorder. An added value of HRVBFB intervention is the fact that it can be easily and affordably implemented in everyday life.

## 1. Introduction

Methamphetamine is a highly addictive central nervous system stimulant. Methamphetamine can be synthesized from ephedrine or pseudoephedrine via a straightforward one-step reduction process [1]. These primary ingredients of methamphetamine can be easily acquired in Taiwan in non-prescription medicine and then converted to the final product. This has resulted in the widespread existence of many ‘mom-and-pop’ laboratories and larger laboratories run by criminal organizations in Taiwan that supply high-purity methamphetamine [2]. These factors have led to an epidemic of methamphetamine use in Taiwan. Use of methamphetamine is associated with a number of health risks, including psychosis and other mental illnesses, cardiovascular and renal dysfunction, infectious disease transmission, and overdose [3,4]. There is, therefore, an urgent need to develop effective treatments for methamphetamine use disorder.

Brain et al. reviewed the effectiveness of pharmacotherapy for the treatment of individuals with methamphetamine use disorder by examining 17 different medications (including antidepressants, antipsychotics, psychostimulants, anticonvulsants, and opioid antagonists) [5]. The results indicated that most drugs evaluated with regards to their merits in treating methamphetamine use disorder were not found to have a statistically significant benefit in terms of abstinence, overall use, and treatment retention. Psychological treatment for psychostimulant use has shown some promising effects in terms of reducing treatment program dropout rates and increasing the number of people achieving continuous abstinence [6]; however, Vocci et al. reviewed previous studies on psychotherapy for stimulant users, and concluded that several operational barriers, such as treatment remaining unaffordable for most patients, and programs requiring intensive staffing, render psychological treatment difficult to implement [7].

Heart rate variability biofeedback (HRVBFB) is an accessible, affordable intervention that has been demonstrated to be of clinical value for the treatment of numerous physical and mental health conditions [8,9,10]. The exact mechanisms of HRVBFB are not entirely clear at this time. Individuals with low heart rate variability (HRV) are usually associated with physical or emotional illnesses [11,12]. Relatively higher levels of HRV have been consistently associated with resilience and individuals’ overall physical health [13,14]. The previous studies also showed individuals with substance use problems have lower HRV than those without substance use problems [15]. HRVBFB arose from observation that increases in the level of heart rate oscillation are produced when individuals use paced breathing to stimulate their cardiovascular system [16]. The levels of heart rate oscillation increase to many times the levels at rest when individuals practice HRVBFB [8,17]. Directly modifying HRV by HRVBFB has shown to be effective in treating mental illnesses with lower HRV [18]. This suggests HRVBFB may have the potential for the treatment of substance use disorders.

HRVBFB treatment is a noninvasive approach for central autonomic networks because the efferent signals from central sympathetic and parasympathetic systems converge on the sinoatrial node [19]. The central autonomic networks play a key role in substance craving by adjusting physiological arousal in accordance with changing situational needs [20]. One study of college students focused on substance users’ cravings and showed that HRVBFB could reduce their cravings for drugs, especially in those with a higher craving at baseline [21]. Penzline et al. revealed that short-term HRVBFB intervention in addition to standard rehabilitation care for individuals with alcohol use disorder could reduce cravings and anxiety [22]; furthermore, they identified an increase in long-term abstinence in subjects with alcohol dependency after using HRVBFB in addition to standard rehabilitation care [23]. These results indicated that HRVBFB may help to reduce cravings and maintain abstinence in substance users. Therefore, we posited that HRVBFB may be an effective complementary therapy to outpatient group therapy for people with methamphetamine use disorder. We then developed a brief HRVBFB protocol, which was modified from the previous study and designed to be easily delivered concurrent with an outpatient methamphetamine use disorder treatment program [24]. Training in the use of HRVBFB provided to subjects was adapted from the protocol developed by Eddie et al. [24]; the modified protocol included four sessions of HRVBFB training, with instructions to practice daily.

The present pilot randomized control study had three primary goals: (1) to quantitatively assess whether HRVBFB reduced methamphetamine craving, the severity of methamphetamine dependence, and the rate of positive methamphetamine urine testing to a greater extent than treatment as usual (TAU) alone in subjects with methamphetamine use disorder participating in an outpatient treatment program; (2) to quantitatively assess whether the effects of HRVBFB persisted after HRVBFB treatment; and (3) to compare the baseline level of craving, severity of dependence, and rate of positive urine testing with the same parameters at the end of the HRVBFB intervention and after a 4-week follow-up period. We hypothesized that HRVBFB would be associated with larger decreases in methamphetamine craving, severity of dependence, and rate of positive urine testing during and after the course of complementary HRVBFB treatment as compared with TAU alone. In addition, we also hypothesized that the baseline level of craving, dependence severity, and rate of positive methamphetamine urine testing were able to predict the status of these outcomes, respectively, after treatment and follow-up.

## 2. Materials and Methods

### 2.1. Participants

All participants were enrolled from an outpatient service in a hospital. The participants met the following criteria: (1) methamphetamine use disorder, as diagnosed according to the DSM-5 [25]; (2) no comorbid use of other substances, except tobacco; (3) no comorbid schizophrenia, major depressive disorder, or bipolar disorder; (4) no cardiovascular, lung, and neurological illnesses; (5) 20-year-old or older male; and (6) completed elementary school or higher. Then, participants who met the inclusion criteria were assigned into two groups in a simple randomization fashion at a 1:1 ratio to receive either HRVBFB intervention or TAU only. This study was approved by the Institutional Review Board of Kaohsiung Medical University (KMUHIRB-SV(II)-20170072). Written informed consent was obtained from each participant prior to the study.

TAU comprised four sessions of cognitive behavioral group psychotherapy focused on addiction over a four-week period. In addition to receiving cognitive behavioral group psychotherapy the same as the TAU-only group, individuals in the HRVBFB group also completed one 60-min session of HRVBFB each week for four weeks. At the beginning of the HRVBFB intervention, participants were orally instructed by researchers to guide their breathing at a given frequency of six cycles per minute following the given pacing stimulus. Then, they were asked to breathe at different breath rates (4.5, 5, 5.5, 6, and 6.5 breaths per minute) in order to determine the resonance frequency. Next, for the HRVBFB component of the training, participants’ instantaneous heart rate and respiration rate data were shown on the screen of the monitoring device. Participants were asked to breathe in a way in which these two rates became as close to synchronous as possible. During the four-week intervention, participants were instructed to practice HRVBFB for two 20-min sessions each day, on their own, using a handheld EmWave biofeedback device provided by the researchers (HeartMath Institute, Boulder Creek, CA, USA).

Participants in the HRVBFB group underwent assessment to record the levels of craving for and severity of dependence on methamphetamine, in addition to a urine test for methamphetamine, prior to the beginning of the first biofeedback session (baseline), immediately after completion of the final biofeedback session, and 4 weeks after the last biofeedback session (end of follow-up). The same evaluations were performed in the TAU-only group at the same time points. Adherence was further facilitated by reminders to follow the study protocol by investigators. In addition to methamphetamine-related subject characteristics, we collected demographic data of the participants in both groups at baseline.

### 2.2. Assessments

#### 2.2.1. Visual Analog Craving Scale (VACS)

The VACS, modified from previous studies [26,27], was used to assess the level of craving in the methamphetamine users. The VACS consists of the following single question: “How much did you crave/desire/want to use methamphetamine in the preceding week?” The level of craving was rated from 0 (not at all) to 100 (very much).

#### 2.2.2. Chinese-Mandarin Version of the Severity of Dependence Scale (SDSch)

The SDSch, which consists of 5 questions, was used to measure the severity of dependence on methamphetamine in the preceding week [28]. The score on the SDSch can range from 0 to 15 [29]; a higher total score indicates a greater severity of dependence.

#### 2.2.3. Urine Drug Test for Methamphetamine

An immunoassay for urine methamphetamine was used as a qualitative test. A positive result indicated that participants used amphetamine.

### 2.3. Statistical Analysis

The baseline characteristics of the participants were analyzed using descriptive statistics and compared between the HRVBFB and TAU-only groups using Student’s *t*-test. Group differences in the levels of severity of methamphetamine dependence and craving for methamphetamine following the intervention and at the end of follow-up were also analyzed using Student’s *t*-test. The results of urine tests at intake, the end of treatment, and the end of the follow-up period were compared between the two groups using the χ_2_ test. In order to determine an interaction between time from intake to the end of treatment/follow-up and group, with regards to severity of dependence, level of craving, and rate of positive methamphetamine urine test following treatment and at the end of follow-up, a generalized estimating equation (GEE) [30] was used. The model for the correlation was autoregressive. The GEE, which is an extension of generalized linear models (GLM) for the analysis of longitudinal data, offers advantages over standard regression techniques in that it enables examination of the relationship between variables at all three time points [31]. Regression analysis was used to examine whether the severity of dependence, level of craving, and rate of positive methamphetamine urine test at intake predicted the same characteristics after treatment and at the end of follow-up, respectively. A two-tailed *p* value of <0.05 indicated statistical significance. The sequential Bonferroni procedure was used to adjust for multiple comparisons [32].

## 3. Results

### 3.1. Participant Demographic and Methamphetamine-Related Characteristics

A total of 64 participants who met the inclusion criteria were enrolled in the study and were randomly allocated into the HRVBFB (32 participants) and TAU only (32 participants) groups. One of the HRVBFB participants and two of the TAU-only participants left the study after the baseline assessment. Age and level of education did not differ between groups (Table 1). There were no differences in the methamphetamine-related characteristics, including duration of methamphetamine use and age at initial methamphetamine use between groups.

The HRVBFB group did not differ with regards to the level of craving, severity of methamphetamine dependence, or rate of positive methamphetamine urine test at entry from the TAU group (Table 2). In addition, the level of craving and severity of dependence and the rate of positive methamphetamine urine testing, were significantly lower in the HRVBFB group than the TAU-only group at the end of the intervention. The rate of positive methamphetamine urine testing was also lower in the HRVBFB group than the TAU group.

### 3.2. The Comparisons of Methamphetamine-Related Characteristics during Intervention and Follow-Up

The level of craving and severity of methamphetamine dependence differed significantly between baseline and the end of follow-up (craving: paired *t*-test = 2.288, *p* = 0.029; dependence severity: paired *t*-test = 4.074, *p* ≤ 0.001) in the HRVBFB group. The rate of positive methamphetamine urine testing for the HRVBFB group was lower at the end of follow-up than at baseline (related samples McNemar test = 9.389, *p* = 0.001). In the TAU group, there were no significant differences in the level of craving (paired *t*-test = −0.334, *p* = 0.741) or severity of methamphetamine dependence (paired *t*-test = −0.992, *p* = 0.330), nor the rate of positive methamphetamine urine testing (related samples McNemar test = 0.125, *p* = 0.727) between baseline and the end of follow-up.

There were significant negative interactions between group and time in the level of craving and severity of methamphetamine dependence during the period of intervention (Table 3). The interaction between group and time for the rate of positive methamphetamine urine testing during the intervention was significantly smaller than one. The interactions between group and time were significantly negative for the level of craving and severity of methamphetamine dependence at the end of follow-up. Meanwhile, the change in the rate of positive methamphetamine urine testing differed between groups during follow-up, as demonstrated by a significant interaction between group and time for the rate of positive methamphetamine urine testing.

### 3.3. The Association of Methamphetamine-Related Characteristics at Intake to Outcomes at the End of Intervention and Follow-Up

Furthermore, the level of craving and severity of methamphetamine dependence at study entry positively predicted the level of craving and severity of dependence, respectively, after intervention and at the end of follow-up (Table 4). A positive methamphetamine urine test at baseline significantly increased the risk of a positive test after the intervention but did not significantly increase the risk at the end of follow-up.

## 4. Discussion

The present study investigated the feasibility and efficacy of a brief HRVBFB intervention to reduce the level of craving, severity of dependence, and status of amphetamine use within the context of an outpatient amphetamine use disorder treatment program. The first important finding was that the individuals in the HRVBFB group had a lower level of craving and a lower severity of amphetamine dependence, in addition to a lower rate of positive amphetamine urine testing, after the intervention and at the end of follow-up. In contrast, there were no significant differences in the level of craving, dependence severity, or rate of positive amphetamine urine testing in the TAU group. Second, the changes in the level of craving, severity of amphetamine dependence, and rate of positive amphetamine urine testing were significantly different between groups during the intervention period. In addition, the individuals in the HRVBFB group exhibited decreased levels of craving, severity of amphetamine dependence, and a lower rate of positive amphetamine urine testing during the follow-up period in comparison with the TAU group. Third, the level of craving and the severity of amphetamine dependence at study entry were able to predict the craving level and dependence severity at the end of the intervention and the end of follow-up, respectively; however, the rate of positive amphetamine urine testing at entry only predicted the rate of positive testing at the end of the intervention.

Our observation of a decreased craving for amphetamine following HRVBFB intervention was in agreement with previous studies of HRVBFB that reported reduced food craving in food cravers and attenuated substance craving in individuals with comorbid mental illnesses [10,33]. Furthermore, the HRVBFB group showed a larger reduction in the level of craving than the TAU group, as evidenced by a significant interaction between time and group during the intervention period. Meanwhile, the observation that the reduction in the level of craving was greater in the HRVBFB group than the TAU group at the end of the follow-up period was encouraging, as the result might indicate that the effectiveness of HRVBFB can persist after the end of the intervention. Therefore, our data support the potential use of HRVBFB as a feasible and effective treatment for reducing amphetamine craving in patients with amphetamine use disorder who attend outpatient services. A neuroimaging study has shown that cravings for methamphetamine is related to activation of brain regions, including the prefrontal cortex and temporal cortex, amygdala, and hippocampus [34]. These brain structures have also been shown to be responsible for cravings in users of substances other than amphetamine and are tasked with regulating the balance between the parasympathetic and sympathetic nervous systems [15]. Furthermore, autonomic imbalances between the sympathetic and parasympathetic systems have been demonstrated to be related to increased cravings [35]. The goal of HRVBFB is to reduce autonomic imbalances by engaging the parasympathetic nervous system and recovery from stress-induced sympathetic responses [36]. Lehrer et al. reported that HRVBFB is a therapeutic intervention that can restore automatic dysfunction through mind-body interactions [13]; however, the exact mechanism of the reduction in cravings by HRVBFB in drug users is not clear. Restoring automatic dysfunction may be one of the possible mechanisms. The reward system consisted of ventral tegmental area (VTA), nucleus accumbens (NAc), medial prefrontal cortex (mPFC), amygdala, insula, and other regions [37]. Dopamine, one of the brain’s neurotransmitters, plays an important role in the reward system. Baltazar et al. showed that mPFC has a crucial role in the brain reward system [38]. Furthermore, the activation of the ventral medial prefrontal cortex (vmPFC) can reduce substance users’ cravings [39]. Andy et al. indicated HRVBFB can increase the functional connections of vmPFC to the reward system, including insula, amygdala, and cingulate cortex. This may be another possible mechanism. Further studies are warranted to explore the association between HRVBFB and neurochemistry of brain.

Leyro et al. conducted a review of previous studies and found that few studies assessed whether HRVBFB led to substance use reduction because most studies employed inpatient and abstinent subjects [40]. The present study provided evidence that the rate of positive amphetamine urine testing and the severity of amphetamine dependence decreased after the HRVBFB intervention, and remained so at the end of the follow-up period. Meanwhile, analysis of the interaction between group and time revealed that the HRVBFB group exhibited significantly greater reductions than the TAU group in the rate of positive amphetamine urine testing and the severity of amphetamine dependence during the period of HRVBFB intervention and at the end of the follow-up period. Our results also implied that the effects of HRVBFB on the rate of amphetamine use and the severity of amphetamine dependence lasted beyond the intervention. This result was in line with a previous study of subjects with alcohol dependency within an inpatient treatment program, which showed a significant difference in long-term abstinence from alcohol one year after receiving HRVBFB [23]. A flexible autonomic nervous system can provide for rapid modulation of physiological states and improve self-control [17,41], and improvement in self-control may decrease substance use behavior [42,43]. In addition, autonomic imbalances in the sympathetic/parasympathetic systems have been shown to be correlated with increased drug use [44,45]. Further studies are warranted to explore whether increasing flexibility and restoring imbalances in autonomic nervous systems is a possible mechanism of the decreasing rate of positive amphetamine urine testing and the reduction in the severity of amphetamine dependence in the HRVBFB group. The level of craving, dependence severity, and positive urine testing for amphetamine at study entry were able to predict the level of craving, severity of dependence, and results of urine testing at the end of the intervention. These results provide important information that could enable identification of individuals at risk of poor treatment outcomes. 

## 5. Limitation

The results of the present study should be interpreted in light of its design, sampling, and measurement limitations. The sample was obtained from a single site, which may have increased selection bias, affecting both the internal and external validity. Participants in this study reported subclinical craving levels and dependence severities; thus, our findings may not be generalizable to all individuals with amphetamine use disorder. In addition, daily HRVBFB practice was assessed using a timeline follow-back method but was not confirmed externally. A larger, population-based sample would yield results generalizable to a broader population of amphetamine users.

## 6. Conclusions

In summary, the findings of the present study support an interpretation of potential added clinical benefit of HRVBFB intervention with regards to reductions in the level of craving, severity of dependence, and amphetamine use, within the context of outpatient treatment. Meanwhile, these clinical benefits may last beyond the end of the HRVBFB intervention. In addition, higher baseline levels of craving for, dependence on, and use of amphetamine were found to be predictive of greater levels of craving, dependence severity, and amphetamine use, respectively, at the end of the intervention. The results support further study of the use of HRVBFB as a complementary therapy within amphetamine use disorder treatment programs using larger samples, verified daily practice logs, and, potentially, additional HRVBFB training sessions.

## Figures and Tables

**Table 1:** Demographic and amphetamine-related characteristics of the case and control groups at study intake.

Variable	Case Mean (SD) N = 31	Control Mean (SD) N = 30	*p*
Age (years)	37.94 (9.07)	36.60 (9.97)	0.586
Education (years)	12.71 (2.95)	13.30 (2.52)	0.405
Age at initial use of amphetamine (years)	31.81 (11.46)	28.50 (8.06)	0.199
Duration of amphetamine use (years)	4.52 (4.29)	4.27 (5.76)	0.848

**Table 2:** Level of craving, amphetamine dependence severity and amphetamine use of the case and control groups at each assessment point.

	Baseline			End of Intervention			End of Follow-Up		
	Case	Control	*p*	Case	Control	*p*	Case	Control	*p*
Craving ^a^, Mean (SD)	18.23 (23.04)	16.17 (23.59)	0.731	7.10 (17.17)	19.33 (25.32)	0.032	7.74 (20.12)	17.17 (23.40)	0.097
Severity of amphetamine dependence ^b^, Mean (SD)	5.39 (2.16)	5.17 (2.68)	0.724	3.81 (2.34)	5.27 (3.22)	0.047	3.42 (2.43)	4.80 (3.04)	0.055
Current amphetamine use ^c^, N (%)	16 (51.61%)	16 (53.33%)	0.893	4 (12.90%)	13 (43.33%)	0.008	2 (6.45%)	14 (46.66%)	<0.001

^a^: measured using the VAS; ^b^: measured using the SDS; ^c^: measured by urine testing.

**Table 3:** Effect of HRVBFB intervention on level of craving, severity of amphetamine dependence, and amphetamine use after the intervention and at the end of follow-up ^a^.

	Craving ^c^				Severity of Amphetamine Dependence ^d^				Current Amphetamine Use ^e^			
	End of Intervention		End of Follow-Up		End of Intervention	End of Follow-Up			End of Intervention		End of Follow-Up	
	Coefficient	*p*	Coefficient	*p*	Coefficient	*p*	Coefficient	*p*	Odds Ratio	*p*	Odds Ratio	*p*
Time from intake to end of intervention or follow-up (weeks)	3.16	0.245	0.50	0.734	0.10	0.836	−0.18	0.313	0.67	0.250	0.88	0.477
Group ^b^	15.90	0.100	5.79	0.439	1.89	0.068	0.84	0.260	4.50	0.168	4.25	0.146
Interaction between Group and Time	−14.30	0.006	−5.74	0.033	−1.68	0.009	−0.80	0.007	0.21	0.033	0.22	0.016

^a^: age as control variable; ^b^: control group as reference; ^c^: measured using the VAS; ^d^: measured using the SDS; ^e^: measured by urine testing.

**Table 4:** Predictors of level of craving, severity of amphetamine dependence, and amphetamine use after the HRVBFB intervention and at the end of follow-up.

	Craving ^b^				Severity of Amphetamine Dependence ^c^				Current Amphetamine Use ^d^			
	End of Intervention		End of Follow-Up		End of Intervention		End of Follow-Up		End of Intervention		End of Follow-Up	
	Coefficient	*p*	Coefficient	*p*	Coefficient	*p*	Coefficient	*p*	Odds Ratio	*p*	Odds Ratio	*p*
Age (years)	0.49	0.320	0.02	0.996	−0.11	0.093	−0.11	0.089	1.04	0.548	1.06	0.430
Education (years)	0.47	0.691	0.56	0.557	−0.18	0.161	−0.06	0.611	0.91	0.535	1.00	0.998
Age at initial use of amphetamine (years)	−0.46	0.321	0.42	0.394	0.10	0.134	0.12	0.062	0.91	0.212	0.87	0.106
Duration of amphetamine use (years)	0.12	0.870	1.38	0.056	0.13	0.150	0.09	0.273	0.93	0.489	0.97	0.783
Group ^a^	−12.11	0.008	−11.67	0.011	−1.91	0.002	−1.82	0.002	0.18	0.021	0.07	0.004
Craving level at study entry	0.49	<0.001	0.39	<0.001								
Level of amphetamine dependence at study entry					0.61	<0.001	0.64	<0.001				
Amphetamine use at study entry									5.59	0.024	2.63	0.207

^a^: control group as reference; ^b^: measured using the VAS; ^c^: measured using the SDS; ^d^: measured by urine testing.

## Data Availability

The data will be available upon reasonable request to the corresponding authors.
